# Multi-omics machine learning classifier and blood transcriptomic signature of Parkinson’s disease

**DOI:** 10.21203/rs.3.rs-6837659/v1

**Published:** 2025-06-20

**Authors:** Xianjun Dong, Ruifeng Hu, Ruoxuan Wang, Jie Yuan, Zechuan Lin, Elizabeth Hutchins, Barry Landin, Zhixiang Liao, Ganqiang Liu, Clemens Scherzer

**Affiliations:** Adams Center of Parkinson’s Disease Research and Department of Neurology, Yale School of Medicine, Yale University; Yale School of Medicine; Brigham and Women’s Hospital, Harvard Medical School; Yale School of Medicine; Yale School of Medicine; Neurogenomics Division, Translational Genomics Research Institute; Technome; Brigham and Women’s Hospital, Harvard Medical School; Sun Yat-Sen University; Yale School of Medicine

## Abstract

Early diagnosis and biomarker discovery to bolster the therapeutic pipeline for Parkinson’s disease (PD) are urgently needed. In this study, we leverage the large-scale, whole-blood total RNA and DNA sequencing data from the Accelerating Medicines Partnership in Parkinson’s Disease (AMP PD) program to identify PD-associated RNAs, including both known genes and novel circular RNAs (circRNA) and enhancer RNAs (eRNAs). Initially, 874 known genes, 783 eRNAs, and 35 circRNAs were found differentially expressed in PD blood in the PPMI cohort (FDR < 0.05). Based on these findings, a novel multi-omics machine learning model was built to predict PD diagnosis with high performance (AUC = 0.89), which was superior to previous models. We further replicated this discovery in an independent PDBP/BioFIND cohort and confirmed 1,111 significant marker genes, including 491 known genes, 599 eRNAs, and 21 circRNAs. Functional enrichment analysis showed that the PD-associated genes are involved in neutrophil activation and degranulation, as well as the TNF-α signaling pathway. By comparing the PD-associated genes in blood with those in human brain dopamine neurons in our BRAINcode cohort, we found only 44 genes (9% of the known genes) showing significant changes with the same direction in both PD brain neurons and PD blood, among which are neuroinflammation-associated genes IKBIP, CXCR2, and NFKBIB. Our findings demonstrated consistently lower SNCA mRNA levels and the increased expression levels of VDR gene in the blood of early-stage PD patients. In summary, this study provides a generally useful computational framework for further biomarker development and early disease prediction. We also delineate a wide spectrum of the known and novel RNAs linked to PD that are detectable in circulating blood cells in a harmonized, large-scale dataset.

Parkinson’s disease (PD) is a progressive neurodegenerative disease, with an estimated global number of patients exceeding 13 million by 2040^1,2^. PD is thought to be caused by combinatorial effects of environmental, epigenetic, and genetic contributions that exert many of their effects through *cis*- and *trans*-acting regulation of transcript abundance^3–6^. Progressive loss of dopamine neurons and an increasing burden of α-synuclein-positive neuronal inclusions (the so-called Lewy bodies) are hallmarks of PD^7,8^. Once PD neuropathology crosses a clinically relevant threshold, movement becomes relentlessly more impaired in PD patients. Biomarkers for early detection and quantitative tracking of disease progression are currently lacking^9,10^. By the time a patient is diagnosed with PD based on today’s clinical criteria (e.g., resting tremor, slow movements, and stiffness), up to 70% of vulnerable dopaminergic neurons have been lost^11^. Therefore, developing a panel of biomarkers for early and accurate diagnosis is urgently needed^12,13^.

Additionally, PD is a slowly progressive and complex genetic disease that likely results from multiple genetic risk variants, each conferring small increases in susceptibility. PD GWASs have revealed thousands of genetic variants whose mutations are associated with disease risk^14^. However, the genetic germline is static and thus cannot be used quantitatively to track disease progression over time using serial measurements. While the strategy of constructing an aggregate measure from multiple individual markers has been fruitful in genetic studies of PD risk, the use of markers spanning multiple modalities (e.g., genetic, transcriptomic, clinical, and imaging-based markers) is needed to maximize its utility^15^, as it is unlikely that a single biomarker will adequately capture the genetic and environmental heterogeneity of PD. Individuals with a high risk of developing PD may show developmental potentials in their transcriptomics profiles before having clinical symptoms, even if they may pass the clinical PD tests^16–18^.

Limited sample size remains one of the main pitfalls in current biomarker studies. A systematic review of published studies using α-synuclein species as a PD biomarker found that 84% of studies included 100 PD patients or fewer^19^. Previous efforts have made several individual cohorts available to study PD biomarkers, including the Michael J. Fox Foundation (MJFF) Parkinson’s Progression Markers Initiative (PPMI)^20,21^, the NINDS Parkinson’s Disease Biomarkers Program (PDBP)^22^, and the MJFF BioFIND study^23^. All data from these efforts are now integrated into the Accelerating Medicines Partnership in Parkinson’s Disease (AMP PD) program, which to date has generated the largest PD longitudinal RNA sequencing data for about 8,500 samples covering more than 3,200 participants with deep clinical phenotype data. A study by Craig et al.^24^ conducted an overview analysis of the PPMI dataset, finding that the neutrophil cell abundance is higher in patients with PD, while lymphocyte cell abundance is lower in patients with PD. They used all time-point samples in their cross-sectional analysis and treated the multiple visits from one subject as independent data points, which may not be representative of early time-point samples. In another study by Makarious et al., the authors built multi-modal machine learning models, which have good performances; however, the case and control samples are unbalanced, and the models achieved a modest, balanced accuracy value of 0.68^13^.

In our work, we leveraged the large-scale, total RNA sequencing baseline data (visit month=0) from the AMP PD to find the most significant differentially expressed RNAs from both known genes (e.g., those annotated mRNAs or lncRNAs in GENCODE) and novel, non-coding RNAs which include circular RNAs (circRNAs) and enhancer RNAs (eRNAs) between PD patients and healthy controls in a defined discovery dataset. We then built multi-gene classifiers using a selective set of PD-associated marker genes, as well as an integrative multi-omics classifier using gene expression data, genetic variant data, and clinical information to achieve better performance. In our study, all models were trained on a discovery dataset and tested on an independent replication dataset to evaluate their respective performance. We also validated PD-associated RNAs in an independent cohort with a secondary method. Enrichment analysis was conducted to find the distinguishable functional pathways or gene ontology terms between PD patients and healthy controls using the replicated differentially expressed protein-coding genes. Finally, we developed and tested innovative multi-omics classifiers which provided a reusable computational framework for PD diagnosis in clinical practices.

## Results

### High-quality, large-scale multi-omics cohort for Parkinson’s disease

This study used data from the AMP PD program, which includes eight cohorts from the PPMI, PDBP, and BioFIND study, with PAXgene-based total RNA-seq data, whole genome sequences, and clinical data available for 3,274 participants (release v2). The PPMI is a longitudinal observational study of 1,923 participants with PD, or at risk for PD, and healthy volunteers, thereby contributing comprehensive clinical and imaging data and biological samples from 33 clinical sites around the world. In the PPMI study, patients in the PD cohort have been diagnosed within two years of enrollment. Importantly, they are de novo patients and as such, not yet taking PD medications, which may confound biomarker analyses. The PDBP is another PD program that collects standardized longitudinal clinical data and biospecimens across all stages of PD. The program has 1,604 participants and was developed to accelerate the discovery of promising new diagnostic and progression biomarkers for PD. The PDBP supports basic, translational, and clinical research through hypothesis testing, target and pathway discovery, biomarker development, and disease modeling. The Fox Investigation for New Discovery of Biomarkers (BioFIND) is an observational clinical study designed to discover and verify biomarkers of PD, which includes blood and cerebrospinal fluid, in 118 well-defined, moderately advanced people with PD and 88 control volunteers. (We obtained permission to access the AMP PD datasets on Google Cloud Storage for the analysis. All information on data collection and data processing procedures from the AMP PD program can be found at.)

**Figure 1:**
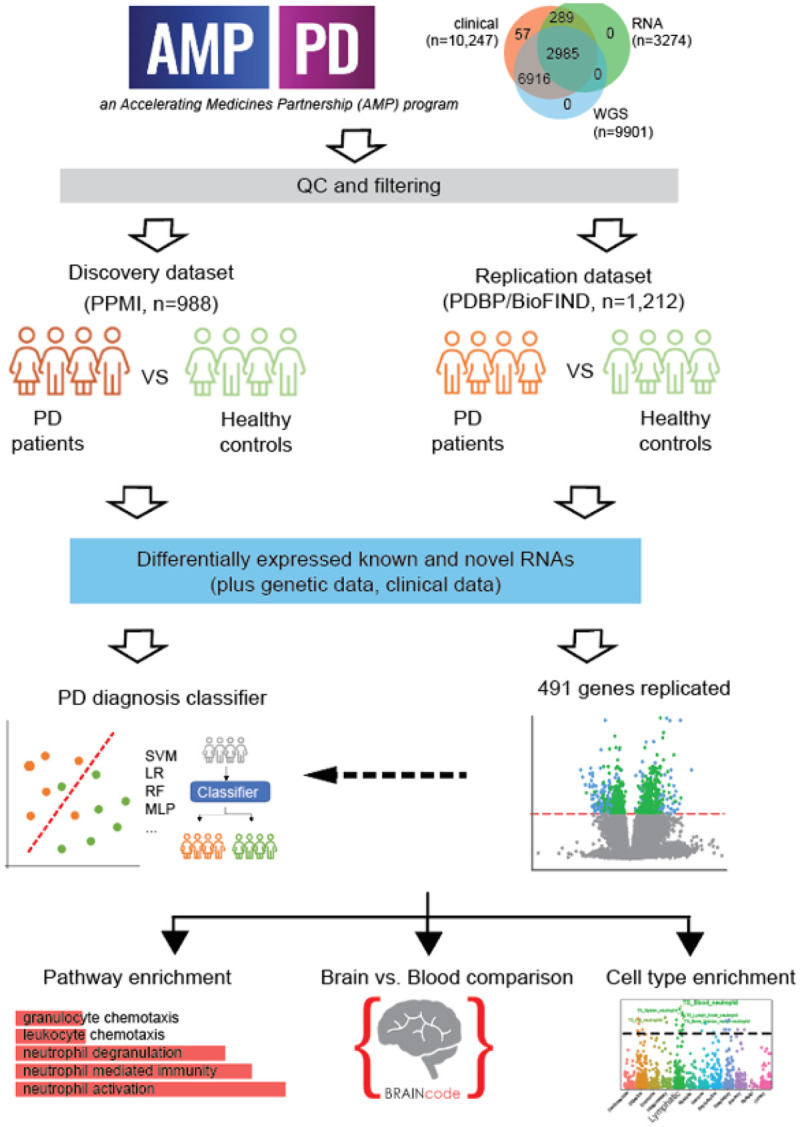
The general workflow of our study. The study was designed in a cross-sectional approach. Significant DEGs/DE eRNAs/DE circRNAs were discovered in PPMI cohorts. The PD diagnosis classifiers were built and tested utilizing the multi-omics data. Then the replicated DEGs/DE eRNAs/DE circRNAs were confirm in the PDBP/BioFIND cohort. Further analysis such as functional enrichment analysis, replication with brain sample data, and cell type enrichment analysis were conducted on the replicated DEGs.

In our study, we leveraged primarily the baseline data (visit month=0) to systematically delineate genes associated with PD diagnosis at an early stage. The PPMI cohort was used as the discovery population and PDBP/BioFIND cohorts were defined as the replication population (Fig. 1). Most of the PD cases in PPMI were recruited when they were newly diagnosed, while most PD cases in the replication dataset were moderate and advanced (**Extended Data Fig. 1**). We wished to establish a set of marker genes exhibiting consistent changes from an early stage of PD and so we intentionally began with a study of individuals recently diagnosed with PD, and then subsequently validated our findings in advanced PD patients. We applied stringent, quality check standards to the samples and the genes (see Methods for details). After the stepwise sample filtrations, 551 PD and 437 control samples remained in the discovery dataset (**Extended Data Fig. 2**), and 760 PD case samples and 452 control samples were used for analysis in the replication dataset (**Extended Data Fig. 3**). After removing low abundance and low variance genes, 35,900 genes remained in the discovery dataset. The sex check shows high consistency between clinically reported sex and genetically determined sex (**Extended Data Fig. 4a**). The case/control sample distributions on the plates showed that the percentages of healthy samples on some plates (P201, P207, P208, P209, P210, P211, P212, P213, P214, and P215) are higher than others in the discovery dataset, but there was no extreme plate outlier (**Extended Data Fig. 4b**). Therefore, we included the plate as a covariant in our analysis design. Based on our data quality assessments, there were no extreme outliers, so we did not remove any additional samples. For the replication dataset, 31,935 genes were kept. The scatter plot demonstrated the sex check consistency between clinically reported sex and genetically determined sex (**Extended Data Fig. 4c**). The case/control sample distributions on the plates show more even distributions across all plates (**Extended Data Fig. 4d**). Finally, 988 baseline samples were included in the discovery dataset and 1,212 baseline samples were included in the replication dataset for all the downstream analyses. The basic clinical characteristics of the datasets after filtrations are summarized in Table 1.

### Over 15,00 known and novel RNAs were found to be differentially expressed in PD blood samples

Through the differential expression (DE) analysis of the GENCODE-annotated human genes in the discovery dataset, 874 known genes were significantly differentially expressed in PD in the discovery dataset with a Benjamin-Hochberg adjusted p-value of < 0.05 (Fig. 1, **Extended Data Table 1**). In this study, we also identified a total of 26,035 candidate eRNAs using our in-house scripts^6^, all of which surpassed the established low expression filtration threshold (only eRNAs with read count > 5 in > 10% samples were kept). Out of these candidate eRNAs, 783 exhibited differential expression. Meanwhile, we identified 441,811 circRNAs in the blood samples. After applying a filtration process requiring at least two read counts in 10% of the samples, 3,052 circRNAs remained and were subjected to DE analysis, which has resulted in a discovery of 35 DE circRNAs (**Extended Data Table 2**). In total, we found 1,692 known and novel RNAs differentially expressed in PD blood.

### Performance of PD diagnosis classifier models

**Figure 2:**
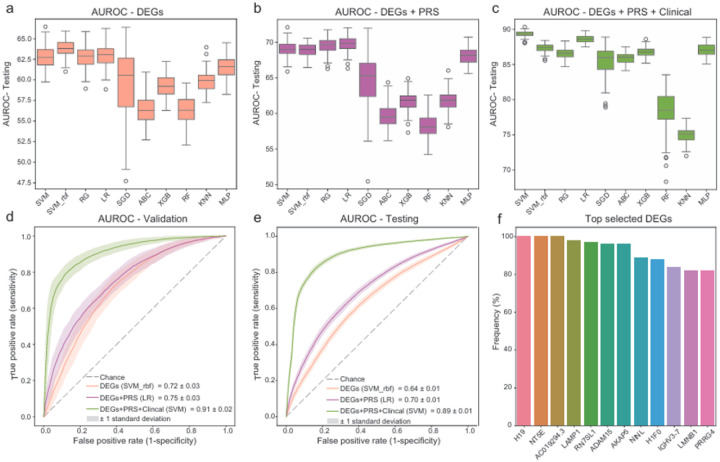
The performance of the models. (a-c) the AUROC value distributions of 10 tested algorithms with step-wised add feature sets on the testing dataset. (d, e) AUROC curves of the best models with step-wised add feature sets on the validation dataset and testing dataset. (f) Top selected genes during feature selection.

Utilizing the identified differentially expressed genes, eRNAs, and circRNAs, we constructed machine learning models for PD diagnosis classification. The PPMI samples (discovery population) were randomly split into a training set (80%) and a validation set (20%) 100 times, so that we had 100 pairs of training-validation datasets. The training set was used to build the classifiers which were validated on the corresponding validation set. All the trained models were tested on the independent replication cohorts. Different machine learning classifiers (n = 10) were trained and compared (see Methods for details). Since there were too many features, and to avoid overfitting problems during the model training, we used LASSO (least absolute shrinkage and selection operator) for feature selection. The PD diagnosis classifiers were first constructed using the 874 DEGs from PPMI with FDR < 0.05. After feature selection, there are 23 to 36 DEGs that were selected as the final predictors from each random split for model training and validation. Among all the 10 tested algorithms, we observed that the support vector machine with RBF kernel (SVM_rbf) had the best performances among others regarding the average area under the receiver operating characteristic curve (AUROC) values and the area under the precision-recall curve (AUPRC) values on the PDBP/BioFIND testing dataset (Fig. 2a). The mean and standard deviation of the AUROC and AUPRC values were 0.72 (0.03) and 0.72 (0.04), respectively, on the PPMI 20% withheld validation datasets, and 0.64 (0.01) and 0.74 (0.01) when the model was applied to the PDBP/BioFIND testing dataset (Table 2, **Extended Data Table 3**). After adding the polygenic risk score (PRS) as the genetics feature to the selected DEGs in each random split, the logistic regression (LR) model demonstrated the best performance as the AUROC and AUPRC were improved to 0.75 (0.03) and 0.78 (0.03), respectively, on the validation datasets, and 0.70 (0.01) and 0.79 (0.01) on the independent testing dataset (Fig. 2B, Table 2). With the clinical data (UPSIT-smell test score, sex, and age) added, the support vector machine (SVM) emerged as the optimal model. The AUROC and AUPRC values were raised to 0.91(0.02) and 0.92(0.02) on the validation dataset, and 0.89 (0.01) and 0.93 (0.01) on the testing dataset (Fig. 2c, Table 2). Fig. 2D and Fig. 2E show the progressive improvement in model performance with stepwise addition of the genetics and clinical features on the validation and testing dataset.

The comparisons of PD prediction potentials using DEGs, DE eRNAs, or DE circRNAs revealed that DE eRNA models exhibited comparable performances to the DE circRNA models, but their predictive powers fall below those of DEG models (**Extended Data Fig. 5, Extended Data Table 3**). Moreover, combining all DE eRNAs, DE circRNAs, and DEGs did not contribute to an enhancement in model performance. Further exploration into the PD prediction abilities of PRS or clinical data demonstrated that the best PRS-based model displayed similar AUROC and AUPRC values to the DEG+PRS model on the testing dataset (Table 2, **Extended Data Table 3**). However, it exhibited lower precision, balanced accuracy values, and notably low specificity values, suggesting a tendency for PRS-based models to yield false positives. Additionally, PRS-based models had inferior performance on validation datasets compared to DEGs-based models. Therefore, DEGs proved effective in compensating for the shortcomings of PRS in predicting PD samples. Clinical data exhibited superior capabilities in distinguishing PD cases from healthy controls, although the performance values slightly lagged our final multi-omics model (Table 2, **Extended Data Table 3**).

Delving into the selected features in each split, a total of 99 genes were chosen, with 12 of them recurrently selected as predictive features more than 80 times out of 100 selections (Fig. 2f **Extended Data Table 3**). The remarkable consistency in feature selection, coupled with low standard deviations in performance values, affirmed the high stability of our model. Looking into those selected genes, *H19* is a long non-coding RNA, which was selected 100 times. H19 has been reported to be associated with PD progression and correlated with susceptibility to various CNS disorders^25,26^. We also found that 7 neutrophil genes were selected as the predictor during the 100 splits, which include *PREX1, SLCO4C1, CXCR2, DNAJC3, CD93, LAMP1*, and *HEBP2*. The *LAMP1* was recurrently selected 98 times. *PREX1* and *CXCR2* were also the two genes that were replicated in brain data.

Above all, our final multi-omics model outperformed the recent publication with similar models with respect to accuracy (0.82 vs 0.75), balanced accuracy (0.83 vs 0.68), and AUROC (0.89 vs 0.85) on the testing dataset (Table 2). We observed better performances than in the previous report, maybe because we included other genes in our DEGs as features in our models, in addition to the protein-coding genes used in the previous study. Additionally, we calculated the PRS using the 7,057 PD-associated significant variants instead of only the 90 SNPs that were used in the previously published paper. Also, the study by Makarious et al., they did not consider the balance issues between positive and negative samples for training the model, that may be also why their specificity value is low. Our performances are more robust since we used balanced training and testing dataset

### Replication of discovered DE RNAs

**Figure 3:**
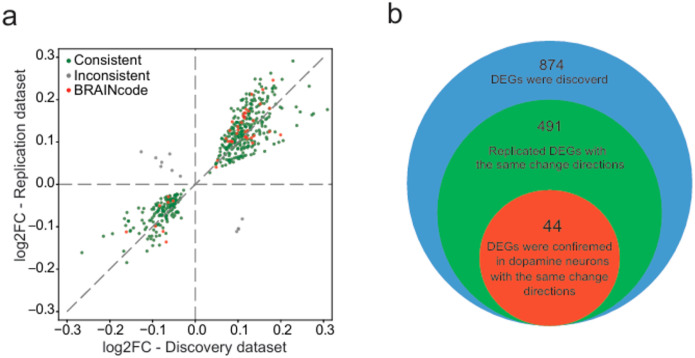
Results of differentially expressed gene analysis. The 502 genes were replicated with a nominal p-value < 0.05 in the replication dataset (the green dots). The scatter plot and the Venn diagram show among the 502 replicated genes, 491 replicated DEGs have consistent change directions in both discovery and replication datasets. Forty-four replicated DEGs with consistent change directions were further confirmed in dopamine neuron samples.

To utilize the potential of the discovery dataset and confirm the discovered DE genes, DE eRNAs, and DE circRNAs, the DE genes and DE RNAs were also called from the replication dataset. Of the 874 DEGs from discovery dataset, 502 genes were replicated in the replication dataset with a nominal p-value < 0.05 (Fig. 3a), of which over 97.8% (491) of the genes have consistent direction changes in both discovery and replication datasets (Fig. 3b, and the details are available in **Extended Data Table 1**). In the 783 initially discovered DE eRNAs, 599 of them were replicated with the same directional changes in the combined PDBP/BioFIND replication dataset. The dataset also revealed that among the replicated DE eRNAs, 396 were up-regulated, and 203 were down-regulated (**Extended Data Table 2**). Regarding circRNAs, 21 of the initially discovered DE circRNAs were replicated in the PDBP/BioFIND dataset. Among these replicated DE circRNAs, 15 were up-regulated, and 6 were down-regulated (**Extended Data Table 2**). The most significant DE eRNA in discovery dataset is chr5_10486550_10486710_plus (This location falls in the region of gene LINC02212), and the most significant DE circRNA in discovery dataset is chr1_17341942_17342402_plus (This location falls in the intron region of gene PADI4).

We then further investigated the host genes of these replicated DE eRNAs and DE circRNAs. The 599 DE eRNAs and 21 DE circRNAs were mapped to 306 host genes (289 eRNA host genes, 18 circRNA host genes, 1 shared host gene (ENSG00000159339, *PADI4*)). Although their host genes did not share the same enriched GO terms with DEGs, we noticed several PD-associated genes or genes that are involved in neutrophil activation in the host gene list. *SPI1* is one of the member genes of GO:0042119 (neutrophil activation). It has been reported that *SPIL1* plays a crucial role in the regulation of the genes relevant to specialized functions of microglia, therefore dysregulation of *SPIL1* might contribute to the establishment or development of PD due to the accumulation of activated microglia^27–29^. *PADI4* is a gene that can positively regulate *TNF*-α and *CCL2* which can lead to the development of neuroinflammation^30,31^. *PADI2* coordinates with *PADI4* to regulate the assembly of the *NLRP3* inflammasome to promote *IL-1β* release. Research also showed that *PADI4* can participate in all aspects of neutrophil extracellular traps (NETs)^32^. Moreover, X-linked dystonia Parkinson’s disease is aggravated by increased levels of *PADI2, PADI4*, and inflammation in the prefrontal cortex and its derived fibroblasts^33^. The circRNA host gene *RHBDD1*, also named RHBDL4, has been implicated in a variety of diseases including Alzheimer’s and Parkinson’s disease, which can cleave amyloid precursor protein inside the cell, causing it to bypass amyloidogenic processing, leading to reduced Aβ levels^34^. This gene had a significant negative log2 fold change in PD patients compared to the health controls in both discovery and replication cohorts. Among the 306 host genes, 53 genes were shared with replicated DEGs. The DEGs *IKBIP, LAMP2*, and *VDR* which are associated with PD and as mentioned above, were also among the host genes.

### Neutrophil activation and immune pathways were upregulated in PD patient blood

**Figure 4:**
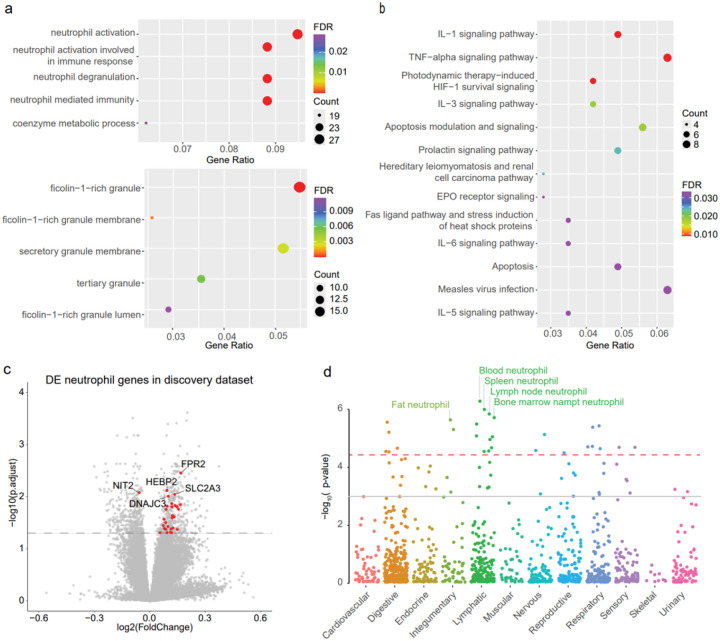
The enrichment analysis results of the replicated differentially expressed genes and the replicated differentially expressed neutrophil genes. (a) The enriched GO-BO and GO-CC terms with adjust p < 0.05, the neutrophil degranulation and neutrophil activation are significantly enriched. And the ficolin-1-rich granule and membrane cell components were significantly enriched. (b) Both the enriched GO-BP and the WikiPathway show that DEGs are involved in immune-related pathways. (c) The volcano plot of replicated differentially expressed neutrophil genes in discovery datasets shows that most neutrophil genes are up-regulated in PD patients (the top 5 ranked genes were shown on the plot, the full list can be found in **Extended Data Table 2**). (d) Cell-type specific enrichment analysis shows the replicated genes are enriched in neutrophil cell types in the lymphatic system. The dashed red line in the plot indicates the significant threshold (p = 3.69 × 10^−5^) corrected with 1355 collected tissue-cell types. The solid grey line indicates the nominal significance (p = 0.001).

The over-representation enrichment analysis was conducted on GO and WikiPathway terms using the 491 replicated genes with the same change directions. There are five significantly enriched GO biological processes (GO-BP) with FDR < 0.05, as well as five significantly enriched GO cellular component (GO-CC) terms (Fig. 4, **Extended Data Table 4**). Both the enriched GO biological processes and cellular components revealed that neutrophil activation and neutrophil degranulation are the key messages derived from the DEGs. Additionally, in the enriched GO-BP terms, we found that the genes were also involved in immune-related pathways, which was also confirmed by the enriched WikiPathways results (Fig. 4b, **Extended Data Table 4**).

By looking into the changes of the leading genes enriched in the neutrophil activation and neutrophil degranulation biological processes, we found that among the 29 DEGs involved in these two GO-BPs, all but one gene were *upregulated* in PD case samples in both the discovery and the replication datasets (Fig. 4c, **Extended Data Table 4**). The results indicate that neutrophil activation and neutrophil degranulation was highly regulated in PD patients. Furthermore, the highly expressed neutrophil genes in up-regulation of the neutrophil activation and neutrophil degranulation pathway can serve as biomarkers for PD early diagnosis.

We then asked on which human tissue and cell types these marker genes might manifest their impacts. By assessing their cell-type-specificity in 1335 curated single-cell and tissue types with WebCSEA, we found that the 491 DEGs were highly enriched in the blood neutrophil cells of the lymphatic organ system (Fig. 4d). This further suggests that the dysregulation of neutrophil cells could be a marker of early PD diagnosis.

Among these 29 differentially expressed leading genes in neutrophil activation and neutrophil degranulation biological processes, several have been studied in the context of PD. A pathogenic mutation (p.N855S) in *DNAJC13* was linked to autosomal dominant Lewy body PD^35–37^. *APAF1* (apoptotic peptidase activating factor) was reported as a potential drug target for neurodegenerative diseases and *APAF1* dominant negative inhibitor can prevent MPTP toxicity as antiapoptotic gene therapy for Parkinson’s disease^38^. *FCGR2A* and *FCGR2B* are well known to play a role in modulating inflammatory responses and to be involved in phagocytosis. Two recent causality analysis of cerebrospinal fluid and blood proteomics showed that *FCGR2A* and *FCGR2B* are among the top causal proteins to PD risk^39,40^. While there is not much evidence for *FCGR2A* and *FCGR2B*’s role in PD blood, Choi et al. showed that *FCGR2B* can function as a receptor for α-syn fibrils and regulate prion-like propagation of α-synuclein in neurons, and the *FCGR2B-SHP-1/−2* signaling pathway may be a therapeutic target for the progression of PD^41^. Lastly, *CD93* participates in pathophysiological processes of central nervous system inflammation^42^.

**Figure 5:**
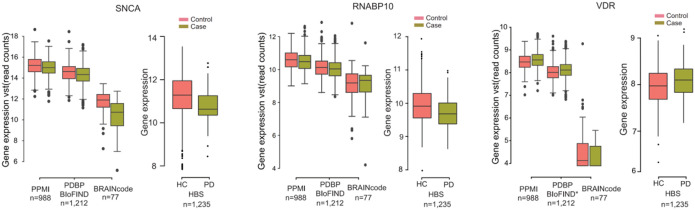
The expression levels of three PD-associated genes *SNCA, RNABP10, and VDR* in the discovery, replication, BRAINcode datasets, and NanoString dataset.

We further validated some of the marker genes with a second digital expression NanoString technology in blood in an independent cohort of the Harvard Biomarker Study (HBS) (Fig. 5, see Methods). *SNCA* is considered as the major causative gene involved in the onset of PD, both from a genetics and protein level^43^. We observed a reduction in SNCA RNA expression in PD samples compared to healthy controls across multiple cohorts, including PPMI, PDBP/BioFIND, BRAINcode, and HBS, using samples from both blood and brain on various platforms, such as RNAseq and NanoString. Our findings consistently showed lower SNCA mRNA levels in the blood of early-stage PD patients, which correlated with brain samples and were consistent with findings in an independent cohort^44,45^. Additionally, we replicated pathological levels of *VDR* and *RANBP10*^46^. Moreover, vitamin D is associated with neuroprotection in animal models of PD^47^ and we previously reported reduced levels of the vitamin D receptor (VDR) in an unbiased microarray screen of PD blood samples^46^ and found a 25-hydroxy-vitamin D deficiency in 17.6% of PD patients^47^. For these previously identified candidate biomarker RNAs of PD, we observed consistent changes in direction between PD and healthy control samples in both the NanoString (HBS) and RNA-seq data (PPMI and PDBP/BioFIND, Fig. 5).

Additional to the dichotomic analysis between the PD and control groups, we further tested if any changes in gene expression are associated with the PD motor severity which is indicated by the MDS-UPDRS part III summary score. Our results are shown in the **Extended Data Table 5**. We found that 2,236 genes and 4,045 genes were significantly associated (adjust p < 0.05) with the MDS-UPDRS part III summary scores in the discovery and replication datasets, respectively. Among these genes, 1,636 genes were shared by both the discovery and the replication datasets with the same change directions. Functional enrichment analysis conducted on the 1,636 replicated genes showed that the “neutrophil activation”, “neutrophil activation involved in immune response”, “neutrophil-mediated immunity”, and “neutrophil degranulation” are the top enriched GO-BP terms. This is consistent with our conclusion from the main dichotomic analysis between the PD cases and healthy controls. These findings suggest that neutrophil degranulation is also a potential biomarker in the blood for PD motor severity.

### Replicating blood-based marker genes in brain neurons

Next, we wondered if any of the marker genes we detected in blood are also presented in brain neurons, as the neuronal RNAs could pass through the blood-brain barrier via mediators (e.g., exosomes) and be detectable in the blood stream. By analyzing the total RNAseq data of dopamine neurons that was laser-captured from >100 human brain samples in the BRAINcode cohort^6^, we identified 575 known genes that were significantly differentially expressed in PD (FDR < 0.05). Compared with the 491 blood marker genes consistently changed in both discovery and replication datasets, 44 genes were further confirmed with the same change direction in dopamine neuron samples (Fig. 3, **Extended Data Table 1**).

Among these 44 brain-blood shared DE genes, the *LAMP2* gene has been reported to be differentially expressed between the early stages of PD and controls, and was also reported to be associated with the expression level of *SNCA*^48^. LAMP2 isoform LAMP2B is also a marker protein expressed on the surface of exosomes, which helps to transport cargos thru the blood-brain barrier. Several neuroinflammation-associated genes were replicated in our brain datasets, such as *IKBIP*^49^, *CXCR2*^50,51^, and *NFKBIB*^52^. Additionally, *IL18R1*, a cytokine receptor that belongs to the interleukin 1 receptor family, was significantly increased in both PD blood and brain neurons. While the function of this cytokine receptor in PD is not experimentally verified, an increase in interleukin-1beta (*IL-1β*) was previously reported as a potential mediator of microglia activation in the PD rat model^53^. These genes were consistently upregulated. Note that only five out of the 44 brain-blood shared genes are in the neutrophil activation and neutrophil degranulation biological processes pathway. They are *PREX1, FCGR2A, CAB39, CXCR2*, and *LAMP2*.

## Discussion

PD is a progressive, multisystem neurodegenerative disease that has been a huge burden on our society and the people it affects. Early diagnosis and biomarker discoveries that bolster the therapeutic pipeline for PD are urgently needed^54,55^. The Accelerating Medicines Partnership in Parkinson’s Disease (AMP PD) program has provided unprecedented opportunities for investigators, including this opportunity, to utilize the data to build an early diagnosis platform for PD patient diagnosis which could lead to improved treatment response and higher efficacy.

Currently, PD diagnosis is mainly based on clinical phenotype detections which can provide high sensitivity for detecting parkinsonism^15,56^. However, clinical observation alone is often insufficient to predict PD status before the onset of the disease. Once symptoms emerge and are detectable, it usually indicates the development of PD(10, 12). It has been reported that in idiopathic PD, there is severe degeneration of the nigrostriatal neurons of the substantia nigra before neurologists can establish the diagnosis according to the widely accepted clinical diagnostic criteria^57^. It is conceivable that neuroprotective therapy starting at such a stage of the disease will fail to stop the degenerative process. Therefore, the identification of patients at risk and earlier stages of the disease appears to be essential for any successful neuroprotection. The observational PD phenotypes are reflections of the changes in transcriptomic profiles which are changing in advance of clinical phenotypes. Analyzing the transcriptomic changes between PD patients and healthy control samples can provide signals for preclinical diagnosis. Utilizing the large cohort datasets from AMP PD in this cross-sectional study, differentially expressed genes were initially discovered and then validated using these large sample-size cohorts.

Functional enrichment analysis was conducted, and we found the neutrophil activation and degranulation were significantly enriched, which we recommend as a diagnostic marker^58^. As previously published, neutrophil infiltration plays an important role in the development of PD^59^. Studies have indicated that circulating neutrophils are increased in number in PD, while other circulating immune cells have either decreased or not changed in prevalence^60,61^. A study by Craig et al that utilized the PPMI dataset found an increased number of neutrophils in PD patients compared to controls^24^. While neutrophils have yet to be identified in the brains of PD patients, neutrophils have been identified in the brains of AD patients and mouse models of neuroinflammation^62,63^. Moreover, circulating neutrophils express *CD11b*, an integrin that responds to aggregated α-synuclein in microglia^64^. Another study revealed that neutrophil degranulation was the most significantly altered molecular pathway in patients, with most genes in the neutrophil degranulation pathway containing nonsense or missense mutations^65^. In our work, we confirmed that the neutrophil activation and degranulation pathway were actively upregulated. By checking the literature and pathway annotation databases^66,67^, we know that neutrophils contain five different types of granules: primary granules, also known as azurophilic granules; secondary granules, also known as specific granules; tertiary granules; secretory vesicles; and ficolin-rich granules. The primary granules are the main storage sites of the most toxic mediators, including elastase, myeloperoxidase, cathepsins, and defensins. The secondary and tertiary granules contain lactoferrin and matrix metalloprotease 9 (also known as gelatinase B), respectively, among other substances. The secretory vesicles in human neutrophils contain human serum albumin, suggesting that they contain extracellular fluid that was derived from the endocytosis of the plasma membrane. Ficolin-rich granules are highly exocytosable, gelatinase-poor granules found in neutrophils and are rich in ficolin-1. Ficolin-1 is released from neutrophil granules by stimulation with fMLP or PMA. Granules are prevented from being released until receptors in the plasma membrane or phagosomal membrane signal to the cytoplasm to activate their movement to the cell membrane for secretion of their contents by degranulation. This is an important control mechanism as the neutrophil is highly enriched in tissue-destructive proteases.

There is increasing evidence showing the links between blood cells and PD development. Variants at, or near, the gene *LRRK2* locus have been known to be associated with PD. Reports have shown that full-length *LRRK2* is a relatively common constituent of human peripheral blood mononuclear cells (PBMC), including affinity-isolated, CD14+ monocytes, CD19+ B-cells, and CD4+ as well as CD8+ T-cells^68^. There was also evidence showing both *SNCA* mRNA and protein are particularly abundant in erythroid cells^4^. Lymphocyte is another category of cells that play important roles in PD. There are enhanced numbers of both CD4+ and CD8+ T cells in the brain parenchyma which had been observed in neuropathological studies of PD^69–71^. A longitudinal case study of a PD patient found that alpha-synuclein-reactive T cells were most abundant in peripheral blood before the appearance of motor symptoms^72^. Above all, more studies are emerging to show the potential of diagnosis biomarkers in the expression profiles of circulating genes.

The TNF-α signaling pathway is another enriched pathway from our WikiPathway enrichment analysis. TNF-α has been proven to be increased both in the brain and in the cerebrospinal fluid of Parkinsonian patients, and TNF-α is involved in the degenerative processes that occur in Parkinson’s disease. TNF-α is the key player in the TNF-α signaling pathway. In our analysis, the leading-edge genes in this pathway include *CFLAR, MAPK3, APAF1, PRKCZ, PYGL, MAP2K4, BTRC, NFKBIB*, and *RAF1*. Currently, there are few studies that have focused on these genes, and so we may study these genes in our future research.

Previous studies have reported an association between the *SNCA* transcript abundance in blood with early stage and imaging-supported, de novo PD. There is a paradoxical reduction in *SNCA* transcript counts in the blood of individuals with early-stage, neuroimaging-supported Parkinson’s disease^4,44,45^. In our analysis, although the *SNCA* transcript abundance did not show significant changes for the patient samples as compared to healthly samples, we confirmed reduced abundance trends in both our discovery and replication cohorts, as well as in our BRAINcode cohort. Literature reports have shown inconclusive SNCA protein changes in plasma which is likely due to hemolysis of erythrocytes in which SNCA is one of the most plentiful proteins^4^.

There have been some studies that established machine learning classifiers with different focuses and using different datasets. Scherzer et al. built the first ML classifier in PD using 22 genes. Liu et al. used clinical and genetic information for the prediction of cognitive decline in patients with Parkinson’s disease and the progression of PD^73,74^, and Severson et al.identified subtypes of PD based on clinical data^75^. Here, to maximize the value of the massive amount of data, we tested several machine-learning methods for PD diagnosis classification using clinical data, transcriptomics data, and genetics data. Our final multi-omics model has high AUC values and high sensitivity and specificity as compared to other reports^13,58^, which means our model cannot only identify the PD patients but also recognize the low-risk individuals. In future studies, we will examine more advanced machine algorithms, such as the DNN, CNN, and VAE, to improve the performances and explore more meaningful insights behind the data.

There may be limitations to the current analysis. The analysis was focused on the diagnostic classification of PD at the baseline in a cross-sectional design. Future analyses will be important to prospectively and longitudinally test diagnostic classifiers. Moreover, progression biomarkers are needed, and this will require analyses of longitudinal RNA data sets. To begin to translate these candidate classifiers to the clinic, more research is needed to clarify the high and low predictive values in different clinically relevant scenarios, for example, as an aid for augmented medicine in the patient populations of movement disorders clinics, or as a screening tool for high-risk individuals in the general population. These scenarios involve highly distinct incidences of PD patients and we require a clearer understanding of high predictive value and low predictive value in the outputs of the models using the selected biomarker genes.

In this study, we identified a set of DE RNAs and defined neutrophil activation and degranulation as potential early diagnostic biomarkers. We built a high-performance PD classification model which could be helpful for PD diagnosis prediction. We provided a computational framework that will be helpful for PD biomarker discovery and provide disease risk prediction, which is a critical step for the better assessment of PD risk and accelerating the diagnosis of Parkinson’s disease.

## Methods

### Study design

First, we discovered genes and RNAs that are differentially expressed in PD in an analysis of the discovery cohort. Also, the novel eRNAs and circRNAs were quantified in both discovery and replication datasets and the significantly differentially expressed eRNAs (DE eRNAs) and circRNAs (DE circRNAs) were presented in this work. Utilizing the DEGs and DE RNAs, genetics, and clinical data, we built the PD diagnosis classifier models for prediction of PD patients. We further replicated those significant DE genes and novel RNAs in a cross-sectional analysis of the replication cohort. In our previous study, we probed the transcriptome of dopamine neurons in post-mortem brains with various levels of neuropathology. We then evaluated the blood-based PD-associated genes (discovered and replicated DEGs) for association with PD neuropathology in dopamine neurons using our laser-captured RNA-seq dataset (BRAINcode,)^6^. Meanwhile, the functional enrichment analysis was conducted on those replicated DEGs. As well, the cell type enrichment analysis was carried out to find the enriched cell types of the replicated DEGs (Fig. 1).

### Sample and gene expression quality control

Filters were applied to remove those participants as shown in **Extended Data Fig. 2** and **Extended Data Fig. 3**. The same filtration strategies were applied to both the discovery and the replication datasets. At the very beginning, participants without RNA-seq were removed. In the next step, only the patients that have the baseline RNA-seq data with RIN greater than 5.0 were kept in our following analysis. To limit batch effects due to ancestry, we restricted our analysis to patients self-identifying as White. Meanwhile, we restricted our analysis to patients listed as either cases or controls. Lastly, we excluded those participants with diagnosis conflicts during the follow-up visits after the initial enrollment in case and control groups separately. Those PD cases whose diagnosis changed during follow-up were removed. Similarly, control participants who developed PD were excluded. Prodromal participants and SWEDD (Scans without evidence of dopaminergic deficit) patients were also removed. Participants with missing clinical or genetic data were also moved as those data would be used in the following analysis.

Quality control of expression data was performed to filter out lowly expressed genes and remove sample outliers. For the genes, we first removed genes that have low expression levels defined as counts of fewer than 5 reads in more than 90% of samples and variances of less than 1 across the samples. To check if there is any sex information that is mislabeled, a scatter plot of the expression levels of a Y chromosome-specific gene and an X chromosome-specific gene was plotted. We also verified the biases of sequencing data arising from case/control sample distributions on the plates were minimal.

### Identification of PD-associated mRNAs

The differential expression analysis was conducted using DEseq2 (v1.36.0)^76^. The gene read counts data from Salmon^77^ quantification result files were used. The primary differential expression was tested between the PD conditions (PD cases vs. healthy controls), and the age_at_baseline (continuous variable), sex, plate, RIN, and the top 10 principal components (PCs) of the genotype data were included as covariates in DEseq2. The replicated DEGs were further analyzed using ClusterProfiler^78^ to find the enriched functions. We also performed cell-type-specific enrichment analysis using the WebCSEA online tool^79^ to find which human tissue-cell types these genes might manifest their impacts on.

As a secondary analysis, we further looked at gene expression changes associated with motor severity, indicated by the MDS-UPDRS part III summary score. Tests were performed in the same DESeq2 framework where the MDS-UPDRS score was treated as a continuous dependent variable.

### Identification of PD-associated enhancer RNAs and circular RNAs

Since the AMP PD provided the raw whole sequencing data, we would like to know the non-coding novel RNAs, especially the eRNA and circRNA differences in PD patients and healthy individuals. We called eRNAs and circRNAs in all datasets. We used our previously developed method^6^ to identify eRNA candidates in the blood. The circRNAs were called using the CIRCexplorer2 package^80^. Then differential expression analysis was conducted on the eRNA and circRNA reads count using DESeq2. Since the circRNAs have relatively lower reads count in the samples, we used all samples, instead of the baseline samples only, to increase the sample size in order to empower the DE circRNA discovery. The same covariates as in finding the DEGs were used.

### Construction of PD diagnosis classifier models

We have built the classifiers utilizing the multi-modality data which includes transcriptomics, polygenic risk score (PRS), and clinical data. The PPMI samples (discovery cohort) were randomly split into a training set (80%) and a validation set (20%). We did the random splits 100 times to test the model’s stability. The training set was used to build the classifiers. The validation set was used to optimize the hyper-parameters of each model through a grid search. Final models were tested on the independent PDBP/BioFIND samples (replication cohort).

Three models were built in sequential order using the following feature sets respectively: transcriptomics only (“DEGs”), transcriptomics plus polygenic risk score (“DEGs+PRS”), and transcriptomics, polygenic risk score, and clinical data combined (“DEGs+PRS+Clinical”). The transcriptomics data is the 874 DEGs from the PPMI cohort. The PRS was calculated using PRSice-2^81^ based on the 7,057 PD-associated significant variants from the recently published PD GWAS work^14^. Clinical data includes the total UPSIT score, sex, and age at the baseline. Since we have too many features, and the feature size is larger than the sample size, we have tried feature selections and modeling the classifiers to avoid overfitting.

To train the models, the variance stabilizing transformed (VST) expression abundances were standardized after log transformations. Feature selection was conducted on the training set using the LASSO approach by making use of sklearn.linear_model.Lasso function and the parameter alpha were screened to pick the best one to have the best area under the receiver operating characteristic curve (AUROC) value. Only features with non-zero coefficients were included in the model. To take advantage of different machine learning algorithms, 10 different machine learning classifiers were trained and compared, including support vector machine with linear kernel (SVM), support vector machine with rbf kernel (SVM_rbf), linear regression (LN), logistic regression (LR), stochastic gradient descent (SGD), AdaBoost classifier (ABC), gradient boosting classifier (GBC), random forest (RF), k-nearest neighbors (KNN), and multiple layers perceptron classifier (MLP).

To investigate if the eRNAs, or circRNAs are predictive for PD diagnosis, we also tested the classifiers using the DE eRNAs, and DE circRNAs separately.

### Confirmation in brain

We tested blood biomarker transcripts using the BRAINcode dataset. The PD-associated RNAs that are also differentially expressed in the brain will be highly relevant and prioritized for validation. We conducted the DE analysis using the data from brain neuron samples and compared the blood DEGs and brain DEGs. In our BRAINcode v2 project, we performed laser-capture microdissection total RNA-sequencing (lcRNAseq)^3^ on dopamine neurons from the midbrain substantia nigra pars compacta of 104 high-quality human postmortem brains (HC: n = 59; ILB: n = 27; PD: n=18). Many polyadenylated and non-polyadenylated transcripts are identified with high confidence. The DEGs in dopamine neurons were identified between PD samples and health control samples. The DEGs with the same fold change directions as in brain data were obtained.

### Evaluation of a second digital gene expression platform in the Harvard Biomarkers Study

We also compared the expression levels of several PD-associated genes from the blood with our in-house NanoString data to validate our findings. The NanoString dataset with PD cases and healthy controls is nested in the Harvard Biomarker Study (HBS). The participant’s blood sample with high RNA quality (RIN ≥ 7) was processed for digital expression analysis on the NanoString platform^82^ with 33 distinct molecular barcodes (29 PD-associated genes) to count the abundance of selected-transcripts directly in RNA from blood cells. A total of 617 PD cases and 618 healthy controls passed normalization processing to validate our findings.

## Supplementary Files

This is a list of supplementary files associated with this preprint. Click to download.

- ExtendedDataFig1.pdf
- ExtendedDataFig2.pdf
- ExtendedDataFig3.pdf
- ExtendedDataFig4.pdf
- ExtendedDataFig5.pdf
- ExtendedDataTable1DEGs.xlsx
- ExtendedDataTable2DEeRNAcircRNA.xlsx
- ExtendedDataTable3Classifier.xlsx
- ExtendedDataTable4Enrichemntanalysis.xlsx
- ExtendedDataTable5UPDRSresults.xlsx

## Figures and Tables

**Table 1.** Summary of clinical data at baseline

		Discovery dataset		Replication dataset		p-value
		PD (*N*=551)	HC (n=437)	PD (n=760)	HC (n=452)	
Sex	Male	318	214	482	204	1.59×10^−4^
	Female	233	223	278	248	0.61
Age, years (mean (sd))		62.91 (10.42)	57.18 (12.53)	64.99 (8.78)	62.94 (10.81)	0.89
Duration of disease (mean (sd))		2.37 (4.12)	\	6.19 (5.18)	\	5.98×10^−41^
RIN value (mean (sd))		8.18 (0.90)	8.36 (0.84)	7.44 (0.83)	7.41 (0.85)	1.0
MOCA (mean (sd))		24.86 (4.17)	27.94 (1.88)	25.30 (3.60)	26.53 (2.54)	1.0
UPSIT (mean (sd))		22.22 (8.03)	33.6 (4.70)	19.62 (7.74)	32.48 (6.00)	0.84

**Table 2.** Performance merits of classifier models on validation and testing datasets

Features (Model)	Validation on PPMI withhold samples							Testing on PDBP/BioFIND samples			
	Accuracy	Sensitivity	Specificity	Precision	BalAcc	AUROC	AUPRC	Accuracy	Sensitivity	Specificity	Precisio
DEGs (SVM_rbf)	0.67 (0.03)*	0.77 (0.04)	0.55 (0.05)	0.69 (0.04)	0.66 (0.03)	0.72 (0.03)	0.72 (0.04)	0.63 (0.01)	0.73 (0.02)	0.47 (0.03)	0.70 (0.01)
PRS (LR)	0.58 (0.03)	0.90 (0.06)	0.17 (0.04)	0.58 (0.03)	0.53 (0.03)	0.54 (0.03)	0.62 (0.04)	0.67 (0.01)	0.94 (0.03)	0.21 (0.07)	0.67 (0.01)
Clinical (SVM)	0.78 (0.03)	0.78 (0.03)	0.79 (0.04)	0.82 (0.04)	0.79 (0.03)	0.85 (0.02)	0.86 (0.03)	0.79 (0.01)	0.76 (0.01)	0.83 (0.01)	0.88 (0.01)
DEGs+ PRS (LR)	0.69 (0.03)	0.74 (0.03)	0.62 (0.05)	0.71 (0.04)	0.68 (0.03)	0.75 (0.03)	0.78 (0.03)	0.66 (0.01)	0.69 (0.02)	0.60 (0.02)	0.74 (0.01)
**DEGs+PRS+Clinical (SVM)**	**0.83 (0.02)**	**0.83 (0.03)**	**0.84 (0.04)**	**0.86 (0.04)**	**0.83 (0.02)**	**0.91 (0.02)**	**0.92 (0.02)**	**0.82 (0.01)**	**0.79 (0.01)**	**0.87 (0.01)**	**0.91 (0.01)**
DEGs+PRS+Clinical (Previous study**)	0.86	0.89	0.76	0.91	0.82	0.90	NA	0.75	0.93	0.43	0.74

*: They are the mean values from the 100 random splits, the values in the parentheses are the standard deviations.

**: The previous study did the independent test on the PDBP dataset.

## Data Availability

The PPMI, PDBP, and BioFIND data can be accessed from the AMP-PD Google Cloud Storage with the approved “Data Use Approvement”. All the up-to-date information and data collection or data processing procedures on the AMP-PD program can be found at https://www.amp-pd.org. The brain neuron data and the NanoString were produced in our own lab. All the analysis code can be accessed at:.
